# Opening or closing eyes at rest modulates the functional connectivity of V1 with default and salience networks

**DOI:** 10.1038/s41598-020-66100-y

**Published:** 2020-06-04

**Authors:** Víctor Costumero, Elisenda Bueichekú, Jesús Adrián-Ventura, César Ávila

**Affiliations:** 1 Center for Brain and Cognition, University Pompeu Fabra, Barcelona, Spain; 2 Neuropsychology and Functional Neuroimaging Group, University Jaume I, Castellón, Spain

**Keywords:** Neural circuits, Visual system

## Abstract

Current evidence suggests that volitional opening or closing of the eyes modulates brain activity and connectivity. However, how the eye state influences the functional connectivity of the primary visual cortex has been poorly investigated. Using the same scanner, fMRI data from two groups of participants similar in age, sex and educational level were acquired. One group (n = 105) performed a resting state with eyes closed, and the other group (n = 63) performed a resting state with eyes open. Seed-based voxel-wise functional connectivity whole-brain analyses were performed to study differences in the connectivity of the primary visual cortex. This region showed higher connectivity with the default mode and sensorimotor networks in the eyes closed group, but higher connectivity with the salience network in the eyes open group. All these findings were replicated using an open source shared dataset. These results suggest that opening or closing the eyes may set brain functional connectivity in an interoceptive or exteroceptive state.

## Introduction

Looking for someone in a crowd, driving to an unfamiliar location, or walking by a place where there could be a dangerous animal on the loose are situations where people keep their eyes wide open. By contrast, the majority of us tend to close our eyes when we are trying to think or remember something. These daily life situations give us clues that, at the brain level, there must be some biological mechanisms that change and adapt when the attentional focus is externally or internally self-driven. These changes might be observable in functional connectivity (FC) networks under minimal experimental manipulation, for instance, during a resting-state condition.

Task-related fMRI evidence has shown that volitional opening or closing of the eyes during non-changing external stimulation leads to two different brain activity configurations: one associated with an “interoceptive” state (with the eyes closed), characterized by activations in areas related to imagination and multisensory activity; and the other associated with an “exteroceptive” state (with the eyes open), characterized by activations in areas related to attention and ocular motor activity^1,2^. This change in brain activity patterns has been shown, independently of light input^3^ and in early-blind individuals^4^, dismissing the possibility that the effects would be driven by exogenous visual stimulation. Furthermore, evidence suggests that it is an instant phenomenon, given that it has been found even during spontaneous eye blinks^5^.

Despite the relevance that this phenomenon may have in FC resting state studies, researchers have used eyes open (EO) and eyes closed (EC) settings indistinctly, with no consensus about which is more suitable depending on the aim of the research. Even more alarming, data show that in January of 2016 about 18% of the most recently published studies did not report the approach used^6^. However, the literature indicating the existence of brain differences in both activity and connectivity based on these conditions is overwhelming. Within the scope of the functional magnetic resonance imaging (fMRI) technique, differences between EO and EC conditions have been shown using a variety of methodological approaches, such as task fMRI^1,2,7,8^, multimodal associations with EEG data^9–13^, spectral analysis derived measures^14–22^, regional homogeneity^15,17,23^, analysis of signal amplitude^24,25^, seed-based FC^15,26,27^, dynamic FC^28^, independent component analysis^29^, time-to-time fluctuations in resting-state^30^, Gaussian Bayesian network analysis^31^, and network derived measures^17,32–35^. Surprisingly, in spite of the large number of methodologies used within this corpus of investigation, there is still a very basic question that has been poorly investigated: *is it possible to observe and characterize the brain FC modulation by the eye condition just by studying the whole-brain FC of the primary visual cortex* (V1)*?*

To the best of our knowledge, no study has been carried out to specifically answer this question. To date, only one study provides direct evidence in this regard^26^; however, the aim pursued in that investigation was different from the aim of the present study, and the evidence was based on a relatively small sample. Specifically, the main objective of that study was to describe the relationship between brain FC at rest and brain local activity by combining fMRI and positron emission tomography techniques. In one of the analyses presented, the authors investigated differences between EC and EO conditions in the whole-brain FC of V1 by comparing two groups of eleven participants each. The results showed increased FC between V1 and salience network areas and the thalamus in the EO group, compared to the EC group. The reverse contrast (EC>EO), however, did not show statistically significant results. Other studies have shown evidence of higher FC between visual areas and the superior parietal gyrus, inferior parietal gyrus, precentral gyrus, and other visual system areas during EO, compared to EC, as well as higher FC between visual areas and the precentral gyrus, postcentral gyrus, superior temporal cortex, and middle temporal cortex during EC, compared to EO^15,35^. Furthermore, another study showed increased effective connectivity from the salience network and central executive network to V1, as well as from V1 to the dorsal attention network, during EO but not during EC^31^. However, again, none of these studies aimed to specifically investigate differences in the FC of V1 due to/related to eye state. On the one hand, one of them aimed to study the reproducibility of resting state data and did not directly study V1, but rather a higher-level processing area in the lateral occipital cortex^15^. On the other hand, the other studies aimed to investigate complex network properties in a specific set of regions, where V1 was one of the many regions that composed the network^31,35^. Thus, it is worthwhile to scientifically gather more evidence about the effects of eye state on the FC of the primary visual cortex. Therefore, the object of the present study was to directly investigate the differences in the FC of V1 in EO and EC conditions by using the simplest and most intuitive resting-state FC approach: seed-based, whole-brain, voxel-wise FC analysis.

Taking into account the existing literature, the main hypotheses of the present study are: 1) EC and EO conditions will modulate the FC of V1; 2) V1 will show positive correlations with areas/networks typically engaged during externally driven tasks, such as the anterior insula and dorsal anterior cingulate (i.e., salience network areas), during EO; 3) V1 will show positive correlations with areas/networks associated with introspective states (i.e. precuneus, inferior parietal cortex, medial frontal cortex) and somatosensory processing (i.e. postcentral gyrus) during EC. To our knowledge, no study has pursued the straightforward objective of exploring the FC of V1 (independently defined and with a whole-brain voxel-wise method) in EC/EO conditions, but its simplicity could shed light on the biological grounds for the internal and external states of the brain.

## Methods

### Participants

A dataset consisting of 198 individuals (95 women; age: mean = 22.2, SD = 4.3, range = 18-40) was collected from various projects of our research group performed with the same scanner. Participants were recruited by placing posters in public places and by word of mouth. Most of them were undergraduate students because our research group is integrated in the campus of Universitat Jaume I of Castellón city (Spain). Three individuals were excluded due to invalid acquisition, and 27 due to excessive head motion. The final sample for the analyses included 168 participants (83 women; age: mean=22.01, SD=4.1, range=18-40). Of them, 63 participants performed a resting-state session in the EO condition (EO-group), and 105 participants completed the resting-state session in the EC condition (EC-group). There were no significant differences in age (t=1.75; p=0.08), gender (χ^2^=0.13; p=0.72), or education level (χ^2^=4.5; p=0.1) between the EO and EC groups (see Table 1 for demographics). All the participants were right-handed according to the Edinburgh Handedness Inventory^36^. None of them had suffered from any neurological or psychiatric disorders, and they had no history of head injury with loss of consciousness. Written informed consent was obtained from all participants, following a protocol approved by the Institutional Review Board of Universitat Jaume I. All the study procedures conformed to the Code of Ethics of the World Medical Association.

**Table 1:** Demographic characteristics and acquisition parameters.

	Test sample		Replication sample
	Eyes Open Group	Eyes Closed Group	Beijing dataset
N	63	105	43
Age (mean ± SD)	21.3 ± 2.04	22.4 ± 4.9	22.7 ± 2.15
Gender (male - female)	52.4–47.6%	49.5–50.5%	48.8–51.2%
Education level	Basic: 0% Middle: 12.3% Superior: 87.7%	Basic: 1.92% Middle: 24.04% Superior: 74.04%	College students
Scanner	Siemens Avanto 1.5 T	Siemens Avanto 1.5 T	Siemens Trio 3 T
fMRI sequence	EPI (24 axial slices; thickness = 4; voxel size = 3.5 ×3.5; TR = 2 s; TE = 48 ms; 200 volumes)	EPI (24 axial slices; thickness = 4; voxel size=3.5 ×3.5; TR = 2 s; TE = 48 ms; 200 volumes)	EPI (33 axial slices; thickness = 3.5; voxel size = 3.125 ×3.125; TR = 2 s; TE = 30 ms; 240 volumes)

SD = standard deviation; TR = repetition time; TE = echo time.

### Image acquisition

Scan sessions consisted of a resting state condition. For the EC sessions, participants were instructed to simply rest with their eyes closed and not sleep or think about anything in particular. The same instructions, but with the specification of keeping their eyes open, were provided in the EO sessions. Just after scanning, participants were explicitly asked if they had followed the instructions and whether they had experienced any issues during the scan. None of the participants reported issues, and they all confirmed that they had followed the instructions. MRI acquisition parameters were the same as those reported in previous studies where we used part of this dataset^37,38^. Images were acquired on a 1.5 T scanner (Siemens Avanto; Erlangen, Germany). Participants were placed in a supine position in the MRI scanner, and their heads were immobilized with cushions to reduce motion. For the resting state fMRI, a total of 200 volumes were recorded using a gradient-echo T2*-weighted echo-planar imaging sequence (TR, 2000 ms; TE, 48 ms; matrix, 64 ×64; voxel size, 3.5 ×3.5 mm; flip angle, 90°; slice thickness, 4 mm; slice gap, 0.8 mm). We acquired 24 interleaved axial slices parallel to the anterior–posterior commissure plane covering the entire brain. Prior to the resting state fMRI sequences, structural images were acquired using a high-resolution T1-weighted MPRAGE sequence (TR/TE = 2200/3.79 ms, flip angle 15°, voxel size = 1 × 1 × 1 mm), which facilitated the localization and co-registration of functional data.

### Image preprocessing

We used the Data Processing & Analysis for Brain Imaging (DAPBI V4.2, http://rfmri.org/dpabi)^39^, to carry out the resting state fMRI data processing. The preprocessing was similar to what was reported in one of our previous studies using this dataset^37^, and it included the following steps: (1) the first five volumes of each raw dataset were discarded to allow for T1 equilibration; (2) slice timing correction for interleaved acquisitions (the middle slice was used as the reference point); (3) head motion correction using a six-parameter (rigid body) linear transformation with a two-pass procedure (registered to the first image and then registered to the mean of the images after the first realignment); (4) co-registration of the individual structural images (T1-weighted MPRAGE) to the mean functional image; (5) Segmentation of structural images into grey matter, white matter, and cerebrospinal fluid using the Diffeomorphic Anatomical Registration Through Exponentiated Lie algebra (DARTEL) tool^40^; (6) removal of spurious variance through linear regression: 24 parameters from the head motion correction (6 head motion parameters, 6 head motion parameters one time point before, and the 12 corresponding squared items)^41^, scrubbing within regression (spike regression as well as 1 back and 2 forward neighbors)^42^ at framewise displacement of (FD)>0.2 mm^43^, linear and quadratic trends, the white matter signal, and the cerebrospinal fluid signal; 7) spatial normalization to the Montreal Neurological Institute (MNI) space (voxel size 3 × 3 × 3 mm); 8) spatial smoothing with a 4 mm FWHM Gaussian Kernel; and 8) band-pass temporal filtering (0.01-0.1 Hz) to reduce the effect of low frequency drift and high frequency noise^44,45^. Given the current debate about the benefits and disadvantages of including global signal regression during preprocessing of FC analyses^46^, we replicated all the analyses, including global signal regression in step 6 described above: “Removal of spurious variance through linear regression”. Furthermore, we also replicated our analyses using the aCompCor method to eliminate physiological noise^47,48^. Specifically, we regressed out the first five components associated with white matter and cerebrospinal fluid signals. The results of these analyses are reported in the supplementary information.

Participants with more than 1.5 mm or 1.5 degree of movement in any of the six directions or less than 120 volumes with FD<0.2 mm (ensuring at least four minutes of rest with low FD) were excluded from the analyses. In average, participants in the final sample showed a mean RMS of 0.12 (SD=0.06, range=0.05-0.7) and a mean FD of 0.14 (SD=0.04, range=0.06-0.25). No significant differences were found between the head motion metrics of the EO and EC groups.

### Functional connectivity analysis

A seed-based correlation approach was used to investigate FC differences between the EO and EC groups. FC estimated with this method relies on the correlation between the average BOLD signal of a region of interest (ROI), also called a seed, and the BOLD signals of other parts of the brain (voxels or other ROIs). For this study, the seed region used was the V1 mask from the HCP-MMP1.0 atlas^49^ projected on MNI space (10.6084/m9.figshare.3501911.v5). This mask was defined in a sample of 210 healthy young adults using a multimodal approach that combined information on the cortical architecture, function, connectivity, and topography (see Supplementary Fig. 1). After ROI definition, a subject-level voxel-wise FC analysis approach was used: for each participant, the correlation was calculated between the time series of the V1 seed and each of the time series of all the voxels of the brain. After the estimation of individual correlation maps, Fisher's r-to-z transformation was performed to normalize correlation values. In order to dismiss the possibility that our results were specific to the selected atlas, we replicated our analyses using different V1 masks. Specifically, we used: (1) the Brodmann Area 17 obtained from the Wake Forest University PickAtlas toolbox (https://www.nitrc.org/projects/wfu_pickatlas/), which is based on the Talairach Daemon database^50^; (2) Brodmann Area 17 obtained from the Anatomy toolbox's maximum probability maps^51^, which is based on cytoarchitectonic information; (3) the maximum probability maps of V1 extracted from the ref. ^52^, which offers a visual cortex parcellation based on retinotopic mapping^52^; and (4) calcarine mask from the Automated Anatomical Labeling (AAL) atlas^53^, which is based on anatomical parcellation of brain sulci. For those parcellations with more than one label for V1 (e.g. left and right), we merged all the V1 masks to make a single bilateral ROI. The results of this analysis are reported in supplementary material.

Group analyses were performed using SPM12 and Matlab R2015b (Mathworks, Inc., Natick, MA, USA). In order to determine the brain regions showing differential FC with V1 seed as a function of resting type, whole-brain, voxel-wise, two-sample t-tests were performed. Age, sex, and mean FD were included as covariates of no interest. The statistical significance threshold was set at p <0.05, FWE-corrected at the cluster level using a voxel-level primary threshold of p <0.001 uncorrected. Given that the results of these analyses may be driven by positive correlations, negative correlations, or a combination of the two, we performed post-hoc tests to further explore our results. The post-hoc tests consisted of voxel-wise, one-sample, t-test comparisons for each group separately, within a mask of the clusters derived from the between-group results. The objective of these post-hoc analyses was merely descriptive, therefore, we set a liberal threshold of p <0.05 uncorrected.

### Replication sample

We used a shared dataset acquired from the open source website http://fcon_1000.projects.nitrc.org/fcpClassic/FcpTable.html (Beijing: Eyes Open Eyes Closed Study), provided by the ‘1000 Functional Connectomes’ Project, to replicate the results of our study. The “Beijing: eyes open and eyes closed” dataset^15^ consists of 48 healthy controls (24 women; age: mean = 22.5, SD = 2.2, range = 18–30) from a community (student) sample from Beijing Normal University in China. Each participant performed three resting state fMRI scans. During the first scan (baseline), participants were instructed to rest with their eyes closed. The second and third scans were randomized between resting types (EO and EC). Table 1 summarizes the MRI acquisition parameters of this dataset. Further details of sample characteristics and image acquisition parameters are reported elsewhere^15^. Data preprocessing and FC estimation were equal to what was reported above, except that we used the prior probability distribution for affine registration of East-Asian brains during segmentation. One participant was excluded due to an incomplete number of volumes in the EO resting condition. Moreover, one participant had an incomplete number of volumes in the EC resting condition. Thus, for this participant, we used the baseline resting session as the EC resting condition. After preprocessing, four participants were excluded due to head motion criteria. Thus, the final sample consisted of 43 participants (see Table 1 for demographics).

The group analyses for this dataset consisted of whole-brain, voxel-wise, paired t-tests comparing the eyes open and eyes closed sessions. The statistical significance threshold was set at p <0.05, FWE-corrected at the cluster level using a voxel-level primary threshold of p <0.001 uncorrected. Post-hoc one-sample t-tests were also performed to further explore our results.

## Results

### Test sample

**Figure 1:**
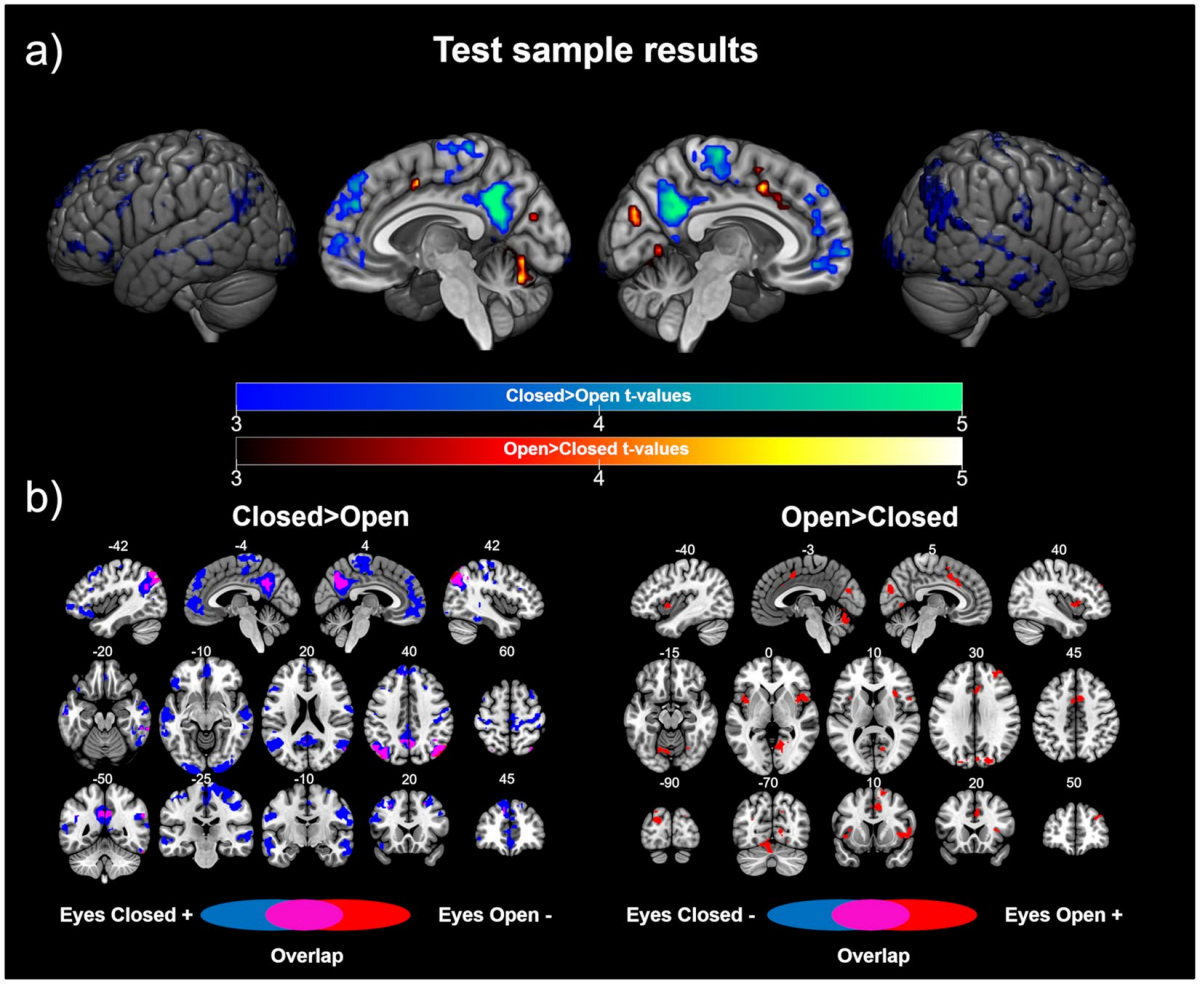
Test sample results: (**a**) Differences in the functional connectivity of V1 according to eye state. Cold colors represent the brain regions showing higher connectivity with V1 in the eyes closed > eyes open contrast. Warm colors represent the brain regions showing higher connectivity with V1 in the eyes open > eyes closed contrast. The color bars represent the t value applicable to the image. (**b**) Contribution of each group to the significant differences. Blue colors represent the brain regions showing a correlation (positive for the contrast eyes closed > eyes open, left panel; negative for the contrast eyes open > eyes closed, right panel) with V1 in the eyes closed group. Red colors represent the brain regions showing a correlation (negative for the contrast eyes closed > eyes open, left panel; positive for the contrast eyes open > eyes closed, right panel) with V1 in the eyes open group. Purple colors represent brain areas showing a shift in the direction of the correlation with V1 according to the eye state. Numbers above slices represents the corresponding MNI coordinate in millimeters.

On the one hand, the results of the analyses investigating the brain regions showing higher FC with V1 in the EC group than the EO group revealed statistically significant differences in two identifiable brain networks, namely, the default mode network, which included the bilateral inferior parietal cortex, ventromedial frontal cortex, and posterior cingulate/precuneus, and the sensorimotor network, which included the bilateral postcentral and precentral gyrus. In addition, we found significant differences in other brain regions, including the bilateral temporal cortex, bilateral inferior occipital cortex, bilateral dorsolateral frontal cortex (superior, middle and inferior gyrus), and dorsomedial frontal cortex (see Table 2 and Fig. 1a). The post-hoc comparisons showed that almost all the differences were driven by positive correlations in the EC group. Interestingly, we found a shift in the FC patterns in V1 as a function of the resting condition in the posterior regions of the default mode network (see Fig. 1b). These results indicated that V1 is correlated with the default mode network during resting with EC, but anticorrelated with this network during resting with EO.

**Table 2:** Brain regions showing significant differences in the functional connectivity with V1 in the test and replication samples.

Region	BA	Test sample			Replication sample		
		MNI (x,y,z)	t value	voxels	MNI (x,y,z)	t value	voxels
***Closed*** > ***Open***							
Posterior cingulate/Precuneus^a^	31	0 -54 27	6.52	447	−3 −57 21	6.06	271
Inferior parietal left^a^	39	−48 −60 30	6.99	367	−39−72 39	5.76	328
Inferior parietal right^a^	39	57 −60 27	6.36	309	42 −66 39	3.85	32
Ventromedial frontal cortex^a^	11	3 45 −12	5.85	196	0 39 −15	5.04	110
Dorsomedial frontal cortex	10	0 51 30	5.67	315	—	—	—
Postcentral/Precentral left^b^	3/4	−54 −9 30	4.82	136	−39 −27 63	7.70	1014
Postcentral/Precentral right^b^	3/4	30 24 66	5.03	427	33 −27 66	7.57	1083
Temporal superior/middle left	21/22	−60 −15 −24	5.75	486	−60 −21 −18	4.78	395
Temporal superior/middle right	21/22	60 −33 −0	6.07	381	63 −15 6	6.26	419
Occipital middle/inferior left	18	−21 −99 −12	5.60	88	−21 −96 −12	6.41	73
Occipital middle/inferior right	18	27 −96 −12	5.90	108	24 −96 −6	5.23	23
Frontal superior/middle left	6/8/9	−24 15 48	4.71	271	−24 3 45	4.78	125
Frontal superior/middle right	6/8/9	48 27 33	5.27	164	15 42 42	5.33	66
Frontal inferior left	45/46/47	−48 30 −9	4.82	170	—	—	—
Frontal inferior right	47	39 33 −15	5.02	25	—	—	—
Hippocampus/Parahippocampus left	—	—	—	—	−21 −12 −21	5.22	71
Hippocampus/Parahippocampus right	—	—	—	—	24 −18 −18	6.07	94
***Open*** > ***Closed***							
Dorsal anterior cingulate^c^	32	9 9 42	5.44	46	12 15 39	4.97	168
Insula left^c^	13	−42 6 0	4.72	26	−36 15 −6	5.55	71
Insula ight^c^	13	36 15 6	4.76	46	42 15 3	4.97	55
Frontal middle left	10	—	—	—	−36 48 33	4.84	86
Frontal middle right	10	33 45 30	5.66	45	33 48 39	5.39	88
Occipital superior/middle left	19	−21 −87 21	5.87	81	−21 −84 18	4.67	118
Occipital superior/middle right	19	21 −84 30	5.04	71	21 −84 30	5.35	116
Cuneus	18/19	6 −81 27	4.64	56	−6 −90 36	5.25	40
Cerebellum	—	−3 −72 −24	4.80	44	−6 −72 −21	7.84	460
Lingual right	19	12 −60 −3	5.20	63	—	—	—
Thalamus	—	—	—	—	18 −18 15	4.87	26
Supplementary motor area	6	15 9 63	4.70	23	—	—	—

MNI coordinates indicate the location of the peak maxima of each region in millimeters. The number of voxels in each region was calculated using xjView toolbox (https://www.alivelearn.net/xjview/). ^a^Brain regions associated to default mode network. ^b^Brain regions associated to sensorimotor network. ^c^Brain regions associated to salience network. BA = Brodmann area.

On the other hand, when we investigated the brain regions showing higher FC with V1 in the EO group, compared to the EC group, we found significant clusters in the bilateral anterior insula and dorsal anterior cingulate cortex, which are the core regions of the salience network. Furthermore, higher FC with V1 in the EO group was found in the cerebellum, bilateral superior occipital cortex, right lingual gyrus, cuneus, right middle frontal gyrus and supplementary motor area (see Table 2 and Fig. 1a). The post-hoc analyses showed that these differences were driven by positive correlations in the EO group.

Similar results were obtained in the analyses that included global signal regression and the aCompCor method during data preprocessing (see Supplementary Fig. 2). Also, the results were similar using the alternative V1 ROIs (see Supplementary Fig. 3).

### Replication sample

**Figure 2:**
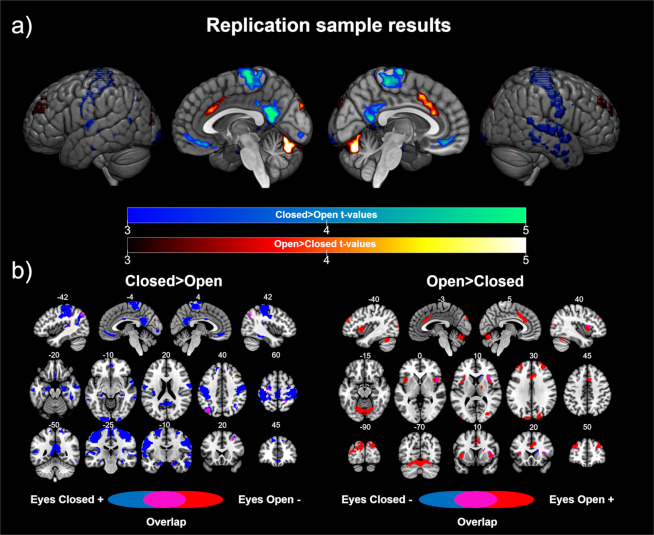
Replication sample results: (**a**) Differences in the functional connectivity of V1 according to eye state. Cold colors represent the brain regions showing higher connectivity with V1 in the eyes closed > eyes open contrast. Warm colors represent the brain regions showing higher connectivity with V1 in the eyes open> eyes closed contrast. The color bars represent the t value applicable to the image. (**b**) Contribution of each condition to the significant differences. Blue colors represent the brain regions showing a correlation (positive for the contrast eyes closed > eyes open, left panel; negative for the contrast eyes open > eyes closed, right panel) with V1 during the eyes closed resting state. Red colors represent the brain regions showing a correlation (negative for the contrast eyes closed > eyes open, left panel; positive for the contrast eyes open > eyes closed, right panel) with V1 during the eyes open resting state. Purple colors represent brain areas showing a shift in the direction of the correlation with V1 according to the eye state. Numbers above slices represents the corresponding MNI coordinate in millimeters.

In order to validate the previously presented results, we replicated our analysis in an independent sample scanned during both EC and EO resting state conditions in the same fMRI session. Using this dataset, on the one hand, we showed significant differences in the sensorimotor network and default mode network areas in the EC> EO contrast, including the bilateral postcentral and precentral gyrus, posterior cingulate/precuneus, ventromedial prefrontal cortex, and bilateral inferior parietal cortex. We also found differences in the bilateral temporal cortex, bilateral inferior occipital gyrus, bilateral superior and middle frontal gyrus, and bilateral hippocampus/parahippocampus (see Table 2 and Fig. 2a). The post-hoc analyses showed that the results were mainly driven by positive correlations with V1 in the EC condition, with a shift in the connectivity patterns in the left inferior parietal cortex (see Fig. 2b).

On the other hand, the results for the EO> EC contrast showed significant differences in the core regions of the salience network (bilateral anterior insula and dorsal anterior cingulate cortex). Furthermore, differences in the cerebellum, cuneus, bilateral superior occipital cortex, bilateral middle frontal gyrus, and thalamus were also found. The post hoc analyses revealed that the differences were driven by positive correlations in the EO condition. Evidence for a shift in the connectivity patterns was found in the right insula.

**Figure 3:**
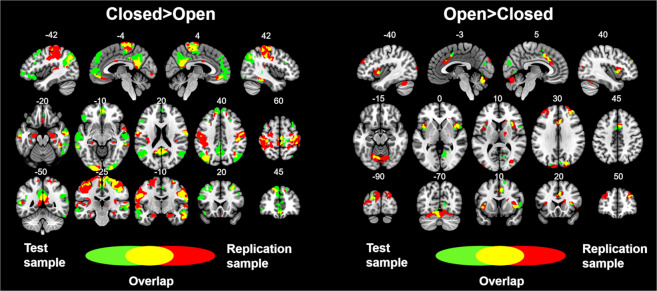
Overlay showing the statistically significant results from the test and replication samples. Green colors show the brain areas revealing significant connectivity with V1 in the test sample. Red colors show the brain areas revealing significant connectivity with V1 in the replication sample. Yellow colors show the brain areas revealing significant connectivity with V1 in both the test and replication samples. Numbers above slices represents the corresponding MNI coordinate in millimeters.

Overall, these results replicated the results observed in the test sample (see Fig. 3). Furthermore, similar results were obtained in the analyses that included global signal regression and the aCompCor method during data preprocessing (see Supplementary Fig. 2), as well as in the analyses using the alternative V1 ROIs (see Supplementary Fig. 3).

## Discussion

The present resting-state fMRI study aimed to investigate the influence that different eye states might have on brain connectivity. The FC of the primary visual area was of particular interest. To carry out this investigation, we used a test dataset consisting of 168 participants and a replication dataset consisting of 43 participants. According to our initial hypotheses, and after performing a whole-brain, voxel-wise, resting-state FC analysis, two main results were found: first, different eye states modulate the connectivity of V1; second, and more importantly, the V1's FC correlation patterns resemble a  “brain's external state” and a “brain's internal state” during EO and EC, respectively. Overall, across the two datasets we found, for the resting-state EO condition, positive correlations between V1 and brain areas typically called the salience network (i.e. bilateral anterior insula and dorsal anterior cingulate cortex)^54^, as well as the superior occipital gyrus, the cuneus, the cerebellum and the right middle frontal gyrus. For the resting-state EC condition, positive correlations were mainly found between V1 and brain areas typically called the default mode network (i.e. Posterior cingulate/Precuneus, bilateral inferior parietal cortex, and ventromedial prefrontal cortex)^55^ and the sensorimotor network (postcentral and precentral gyrus). Furthermore, positive correlations between V1 and the bilateral temporal cortex, bilateral inferior occipital cortex, and bilateral superior and middle frontal gyrus were also found during EC (see Fig. 3 and Table 2).

Our results are consistent with previous investigations showing that the different eye conditions modulate FC during resting-state acquisitions^15,26,31,35^. The closest and most comparable research to the present study is the one conducted by Riedl et al. in 2014. In this study, the authors showed that EO increased the local activity in V1, the secondary visual cortex, and the salience network regions more than EC, as evidenced by whole-brain analysis of positron emission tomography data. In parallel, resting state fMRI demonstrated an increased FC between these regions by combining visual areas and salience network areas into two seeds. As we have replicated here, the authors found that during EO, the primary visual area is more coupled with salience network areas than during EC. However, we expand these results in our study by showing that EC, compared to EO, yielded increased FC between V1 and the default mode network and the sensorimotor network.

The salience network^54^ has been functionally related to the processing of salient inputs^54,56,57^. Indeed, it has been proposed that the integration of the external sensory information and the internal body signals, including emotional information, is carried out by the salience network^54,57^. Some authors have found that the right dorsal anterior insular cortex, a component of the salience network, is responsible for generating control signals that influence the activity of the central executive network and the default mode network^57,58^. This same region is functionally engaged when processing high-demanding attention and control tasks, including evaluation of error, by exerting inhibition or task switching^59^. Thus, the salience network, and especially the dorsal anterior insular cortex, has been suggested as a brain switch from an introspective or internal state (e.g., default mode network) to a readiness to respond state or external state (e.g., salience and central executive network)^57^.

The default mode network has mostly been associated with self-referential processing, moral information processing, and autobiographical and episodic memory retrieval^60–63^. Other introspective or internal functions have been related to the activity of this network, such as the mind-wandering state and future self-imagination^60^. The default mode network has been specifically investigated in the context of eye state. A previous study showed increased FC and amplitude of low frequency fluctuation (ALFF) in the precuneus and medial prefrontal cortex during EO, compared to EC^18^. Moreover, other studies that combined EEG and fMRI data to investigate differences between EC and EO have implicated areas of the default mode network. One study found negative correlations between alertness, measured as the ratio of power in the alpha band over the power in the delta and theta bands, and the activity of the posterior cingulate gyrus, temporal gyrus, and medial frontal gyrus during resting state in the EC condition, compared to the EO condition^10^. By contrast, another study found positive correlations between the alpha power and the default mode network areas in the EO condition^13^. Notably, the study by Falahpour *et al*.^10^ also showed higher positive correlations between alertness and the activity in the thalamus and insula in the EC condition when compared to the EO condition. Taking into account that alpha power is primarily recorded in occipital regions, these results are consistent with the differential pattern in the connectivity of V1 during EC and EO shown in our study.

Together, the connectivity patterns shown in our study agree with the proposal of two brain configurations associated with EO and EC, respectively: an exteroceptive state associated with attention and vigilance and an interoceptive state associated with mental imagery^1,2^. Thus, the fact that the primary visual cortex was highly coupled with the salience network seems to indicate that, in the EO condition, the brain was in an “external mode” or preparing to respond to an expected visual stimulus. Moreover, the connectivity pattern of V1 during the cancellation of visual input in the EC condition may involve a brain configuration that facilitates introspection and mental simulation, processes that have been related to activation in the default mode network and primary sensory cortex^62,64^.

In our opinion, the implications of these results are important for establishing experimental fMRI analysis. On the one hand, performing resting-state fMRI acquisitions in EO conditions establishes by default that at least primary visual areas will be more synchronized with salience network areas. On the other hand, resting-state fMRI acquisitions in EC have, by default, more synchronized connectivity between visual networks and the default mode network, among others. These differences in functional synchronization may be relevant not only for resting state studies, but also to set the control condition in fMRI task studies, given the data indicating that local activity is closely related to FC in EC and EO conditions^26^. In this regard, our results, combined with the results of previous studies investigating the modulation of ALFF at rest based on eye state, may provide further evidence for this relationship, given that increased ALFF in the somatosensory cortex has been consistently reported during EC^14,15,19–22^.

Finally, it should be noted that we found some differences in the results from the test and replication samples. Overall, the test sample showed greater differences in default mode areas (larger clusters and more significant peaks), and the replication sample showed greater differences in sensorimotor regions in the EC > EO contrast. In this same contrast, the test sample showed specific differences in inferior frontal areas and the dorsomedial frontal cortex, whereas the replication sample showed specific differences in the hippocampus and parahippocampus. Moreover, in the EO > EC contrast, the test sample showed specific differences in the supplementary motor area and right lingual gyrus, whereas the replication sample showed specific differences in the left middle frontal cortex and thalamus. Given the many differences between the two samples (one recruited in Spain and the other in China), it is not possible to establish the origin of these differences. Any differences could stem from methodological factors (different design, scanner, and acquisition parameters), environmental factors (different cultures), genetic factors (different ethnicity), or a combination of them. Therefore, researchers should be cautious when interpreting sample-specific results of this study in the context of the eye state.

In summary, in this study, using two independent datasets, we have consistently shown that the FC of V1 is modulated by the resting-state eye condition. Thus, V1 showed positive FC with the salience network during the EO condition. Otherwise, V1 was positively coupled with the default mode network and sensorimotor network during EC. These results suggest that opening and closing the eyes leads to exteroceptive and interoceptive brain configurations.

## Supplementary information

Supplementary Material.

## Data Availability

The data that support the findings of this study are available from the corresponding author upon reasonable request.
