# Supplementary material for: Opening or closing eyes at rest modulates the functional connectivity of V1 with default and salience networks
Source: Sci Rep. 2020 Jun 4;10:9137. doi: 10.1038/s41598-020-66100-y (PMC7272628; doi:10.1038/s41598-020-66100-y)
Supplement: Supplementary file 1 — Supplementary Material. [file 41598_2020_66100_MOESM1_ESM.pdf]

Opening or closing eyes at rest modulates the functional connectivity of V1 with default and salience networks

Víctor Costumero[1, 2*], Elisenda Bueichekú[2], Jesús Adrián-Ventura[2], and Cesar Ávila[2].

[1] Center for Brain and Cognition, University Pompeu Fabra, Barcelona, Spain

[2]Neuropsychology and Functional Neuroimaging Group, University Jaume I, Castellón, Spain

*Correspondence to vcostume@uji.es

Supplementary figures

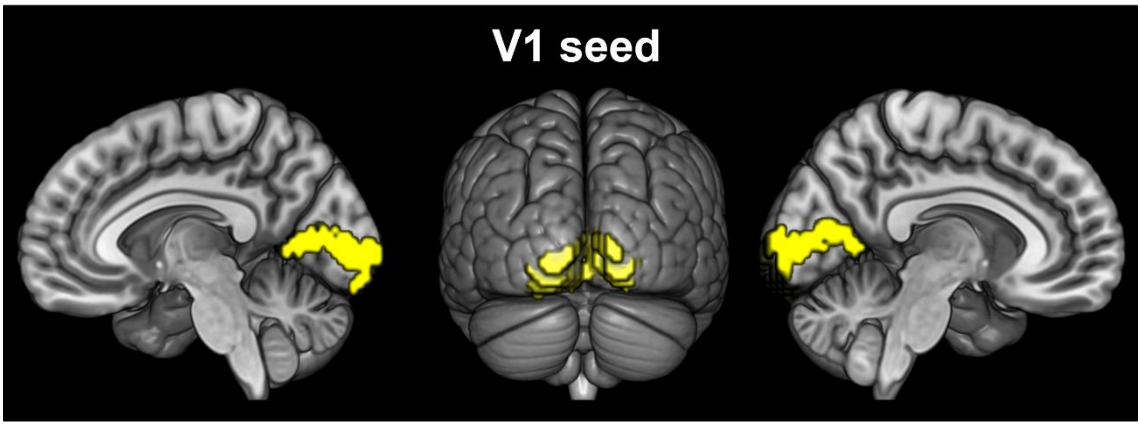

Supplementary figure 1. Sagittal and posterior view of the V1 mask from the HCP-MMP1.0 atlas used as the seed region for functional connectivity analyses.

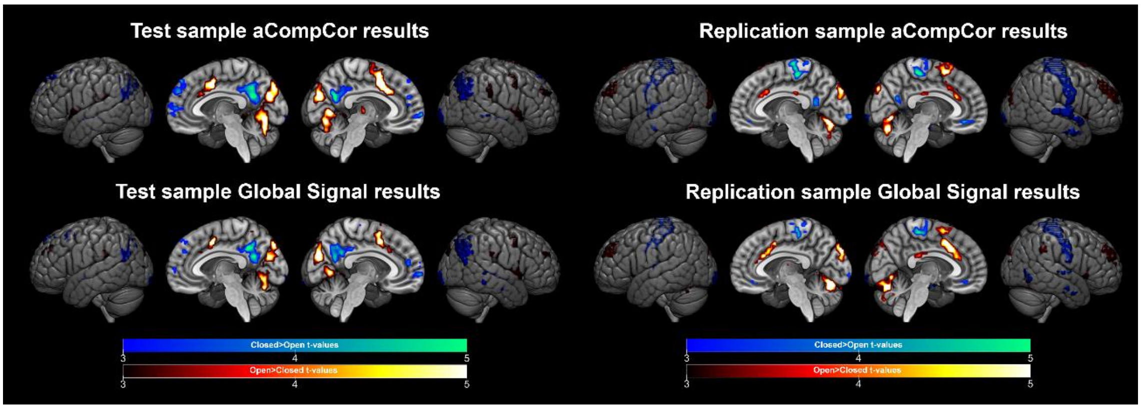

Supplementary figure 2. Results using the aCompCor method (upper panel) and global signal regression (lower panel) during preprocessing for test (left) and replication (right) samples. Cold colors represent the brain regions showing higher connectivity with V1 in the eyes closed > eyes open contrast. Warm colors represent the brain regions showing higher connectivity with V1 in the eyes open > eyes closed contrast. The color bars represent the t value applicable to the image.

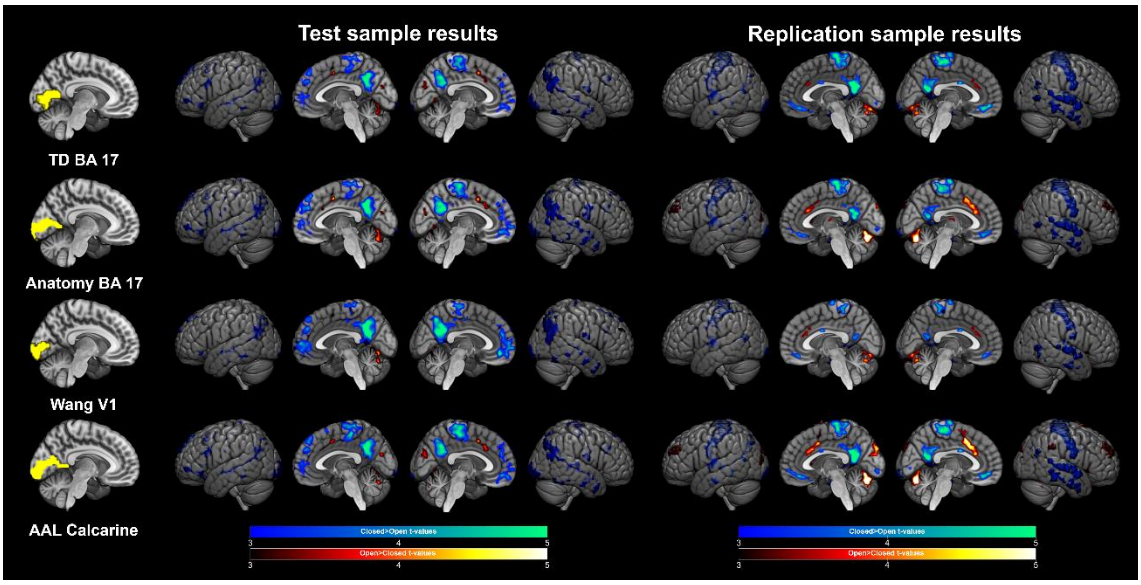

Supplementary figure 3. Results using different ROI definitions for V1. The first row shows the results using the Brodmann Area 17 from the Talairach Daemon database as implemented in the Wake Forest University PickAtlas toolbox (TD BA 17). The second row shows the results using Brodmann Area 17 from the maximum probability maps incorporated in the Anatomy toolbox (Anatomy BA 17). The third row shows the results using maximum probability maps of V1 region reported in the visual cortex parcellation of Wang et al., (2015). The fourth row shows the results using the calcarine mask from the Automated Anatomical Labeling atlas (AAL calcarine). Cold colors represent the brain regions showing higher connectivity with V1 in the eyes closed > eyes open contrast. Warm colors represent the brain regions showing higher connectivity with V1 in the eyes open > eyes closed contrast. The color bars represent the t value applicable to the image.
